# A facile process for adipic acid production in high yield by oxidation of 1,6-hexanediol using the resting cells of *Gluconobacter oxydans*

**DOI:** 10.1186/s12934-022-01947-6

**Published:** 2022-10-28

**Authors:** Sang-Hyun Pyo, Mahmoud Sayed, Oliver Englund Örn, Jorge Amorrortu Gallo, Nídia Fernandez Ros, Rajni Hatti-Kaul

**Affiliations:** 1 Division of Biotechnology, Department of Chemistry, Center for Chemistry and Chemical Engineering, Lund University, 22100 Lund, Sweden; 2 Department of Botany and Microbiology, Faculty of Science, South Valley University, Qena, 83523 Egypt

**Keywords:** Adipic acid, 1,6-hexanediol, *Gluconobacter oxidation*, Polymer building block, Whole cell oxidation

## Abstract

**Background:**

Adipic acid (AA) is one of the most important industrial chemicals used mainly for the production of Nylon 6,6 but also for making polyurethanes, plasticizers, and unsaturated polyester resins, and more recently as a component in the biodegradable polyester poly(butylene adipate terephthalate) (PBAT). The main route for AA production utilizes benzene as feedstock and generates copious amounts of the greenhouse gas NO_2_. Hence, alternative clean production routes for AA from renewable bio-based feedstock are drawing increasing attention. We have earlier reported the potential of *Gluconobacter oxydans* cells to oxidize 1,6-hexanediol, a potentially biobased diol to AA.

**Results:**

The present report involves a study on the effect of different parameters on the microbial transformation of 1,6-hexanediol to adipic acid, and subsequently testing the process on a larger lab scale for achieving maximal conversion and yield. Comparison of three wild-type strains of *G. oxydans* DSM50049, DSM2003, and DSM2343 for the whole-cell biotransformation of 10 g/L 1,6-hexanediol to adipic acid in batch mode at pH 7 and 30 °C led to the selection of *G. oxydans* DSM50049, which showed 100% conversion of the substrate with over 99% yield of adipic acid in 30 h. An increase in the concentrations of the substrate decreased the degree of conversion, while the product up to 25 g/L in batch and 40 g/L in fed-batch showed no inhibition on the conversion. Moreover, controlling the pH of the reaction at 5–5.5 was required for the cascade oxidation reactions to work. Cell recycling for the biotransformation resulted in a significant decrease in activity during the third cycle. Meanwhile, the fed-batch mode of transformation by intermittent addition of 1,6-hexanediol (30 g in total) in 1 L scale resulted in complete conversion with over 99% yield of adipic acid (approximately 37 g/L). The product was recovered in a pure form using downstream steps without the use of any solvent.

**Conclusion:**

A facile, efficient microbial process for oxidation of 1,6-hexanediol to adipic acid, having potential for scale up was demonstrated. The entire process is performed in aqueous medium at ambient temperatures with minimal greenhouse gas emissions. The enzymes involved in catalyzing the oxidation steps are currently being identified.

**Supplementary Information:**

The online version contains supplementary material available at 10.1186/s12934-022-01947-6.

## Background

Replacing the traditional petrochemical processes for chemical production by alternative routes based on renewable feedstock is drawing increasing attention, as the impact of fossil-based greenhouse gas (GHG) emissions on climate change is becoming a global concern [1–5]. Adipic acid (AA) is one of the most important industrial chemicals with a global market estimated at about 5.45 billion USD in 2021 [6]. The majority of AA (61%) is used for Nylon 6,6 and nylon 6 production, while other uses include polyurethanes, plasticizers, unsaturated polyester resins, wet strength paper resins, coatings, synthetic lubricants, and food additives. More recently, AA has been used for biodegradable polyester poly(butylene adipate terephthalate) (PBAT) [7]. AA is thus regarded as the most valuable aliphatic dicarboxylic acid [8].

Most of the global AA production occurs by oxidation of ketone-alcohol oil (KA oil) (obtained from benzene as a starting material) using a high concentration of nitric acid as an oxidant in a process releasing large amounts of NO_2_ as a by-product, which has a contribution of 10% of the global anthropogenic GHG emissions (Additional file 1: Scheme S1) [9, 10]. A method for synthesis of AA via oxidation of adipic aldehyde diacetal obtained by double-n-selective hydroformylation of 1,3-butadiene has been described with reaction yields of around 70% [11]. Various routes for producing biobased adipic acid are being developed at lab scale as well by several companies using a combination of biotechnology and chemical catalysis [12–17]. One of the approaches involves the production of precursor molecules like glucaric acid or *cis,cis*-muconic acid from sugar (and also lignin in the latter case). Glucaric acid is converted to adipic acid by hydrodeoxygenation while muconic acid is processed through Pt-catalyzed hydrogenation [11–13] or via the enzyme enoate reductase [14]. Several metabolic engineering approaches have been proposed for AA production from sugars and lipids [18, 19]. Examples include the elongation of succinic acid with acetyl-CoA to form 3-oxoadipyl-CoA, followed by reverse gamma oxidation to give AA [12, 14, 15], and fatty acid synthesis through β- and ω-oxidation pathway that in an industrial yeast strain is selectively terminated at AA [17]. But all these routes either struggle with low yield, expensive recovery routes and/or substrate costs. Gilkey et al. reported the synthesis of AA from tetrahydrofuran-2,5-dicarboxylic acid (prepared from 2,5-furandicarboxylic acid, FDCA) by metal-free selective hydrogenolysis with up to 89% yield [20, 21].

An alternative source of AA that has recently been described is through oxidation of 1,6-hexanediol (1,6-HD), which can be derived from bio-based hydroxymethylfurfural (5-HMF) [20, 22, 23]. Production of 1,6-HD by hydrogenolysis of HMF using double layered catalysts, Pd/SiO_2_ and Ir–ReOx/SiO_2_ (with 57.8% yield) [24], and Pd/ZrP (43% yield) [25], and by hydrogenation of HMF derivative, tetrahydrofuran dimethanol using Pt-WOx/TiO_2_ (70% yield) has been reported [26]. Chemical oxidation of 1,6-HD using Au as catalyst gives high AA yield but the process requires alkaline conditions followed by neutralization for the product recovery, hence leading to increased cost and waste formation [22]. The use of Pt as a catalyst has shown promise as it does not require a base but undergoes deactivation [23]. In general, low selectivity and toxicity of the catalyst, and harsh conditions required for both upstream and downstream processes present major challenges for chemical synthesis.

The biocatalytic oxidation offers a more selective and mild alternative to chemical processes, and has received attention as a synthetic route [27]. Since whole cells provide a more protective environment for the enzymes, allow regeneration of cofactors, and are also less expensive, the use of whole cell oxidation is generally more preferable to pure enzymes whenever possible [28, 29]. Recently, we proposed a green route for the production of 6-carbon polymer building blocks including AA from 1,6-HD (Additional file 1: Scheme S2) [20]. Complete oxidation of 84.6 mM (10 g/L) 1,6-HD to adipic acid with over 99% yield was achieved using the acetic acid bacteria *Gluconobacter oxydans.*

**Scheme 1:**
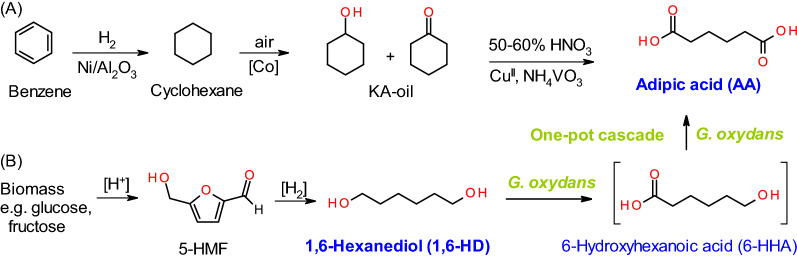
Synthesis of adipic acid by **A** conventional catalytic process by oxidation of KA-oil (ketone-alcohol oil) obtained from cyclohexane derived from fossil benzene, and **B** biocatalytic oxidation of 1,6-hexanediol (potentially produced from biobased 5-HMF) via 6-hydroxyhexanoic acid

In this report, we have extended the study to determine the effect of different parameters, including *G. oxydans* strains, pH, aeration, substrate/product concentration, on the biotransformation, and furthermore demonstrate fed-batch transformation using whole cells and downstream processing of the product as the potential scalable route for adipic acid production (Scheme 1B).

## Results and discussion

Acetic acid bacteria, such as *Acetobacter* and *Gluconobacter*, comprise highly versatile organisms able to produce a variety of compounds used in chemical, medical, pharmaceutical, engineering food and beverage sectors [30]. *Gluconobacter* genus has shown promise for large scale oxidation of a variety of sugars, alcohols and related compounds [30–32]. Selective oxidation of primary alcohols to corresponding carboxylic acids via aldehydes as intermediates, and of secondary alcohols to the corresponding ketones or carboxylic acids using this group of bacteria has been extensively investigated [30, 33–38]*.* Their oxidation abilities have been attributed to a wide range of membrane-bound and cytoplasmic oxidoreductases including alcohol- (ADH) and aldehyde dehydrogenases (ALDH) and oxidases [20, 30].

### Oxidation of 1,6-HD to AA using different *G. oxydans* strains

**Fig. 1:**
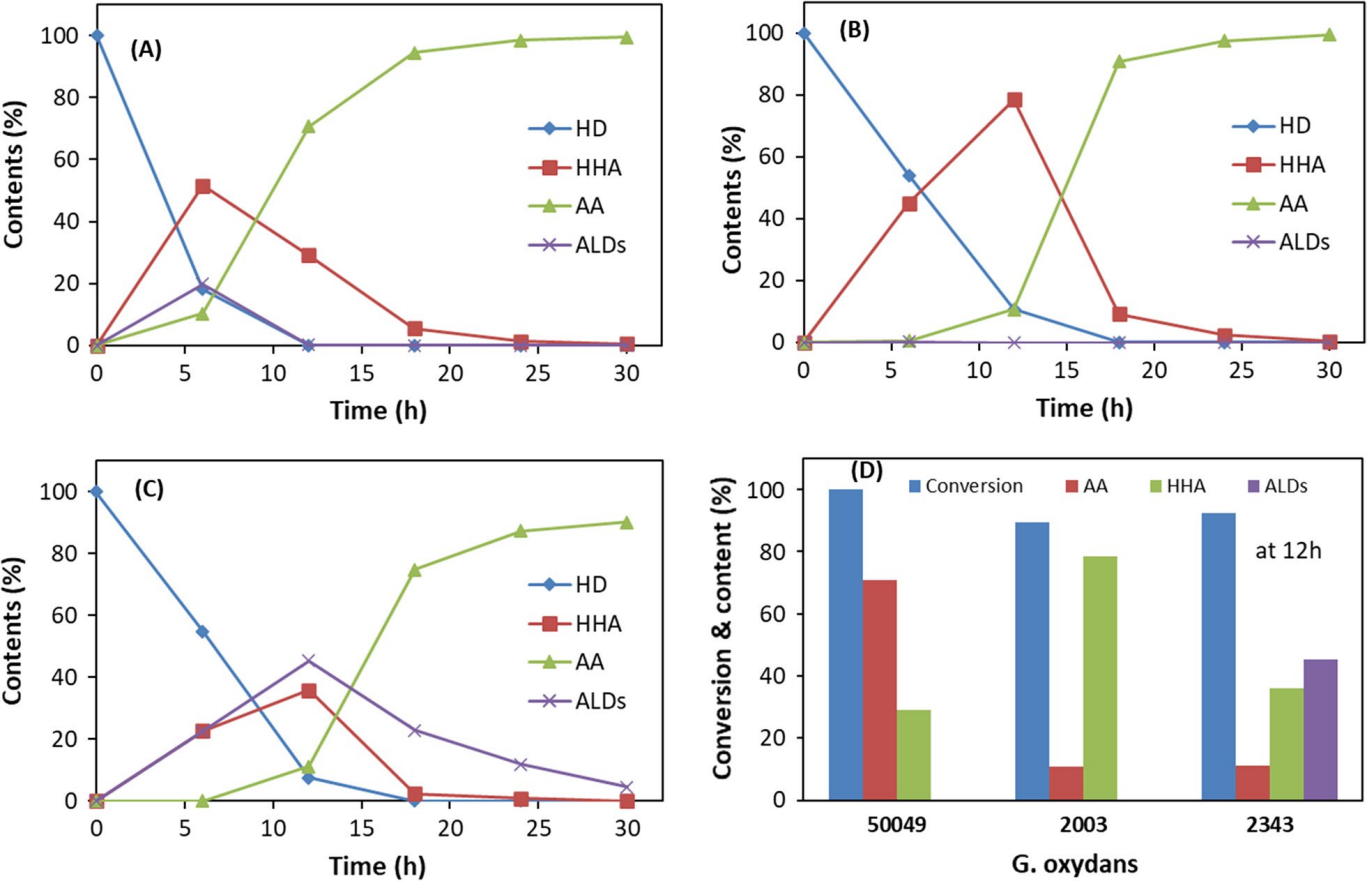
Oxidation of 10 g/L 1,6-HD to adipic acid via 6-HHA by **A** *G. oxydans* 50049, **B** *G. oxydans* 2003, **C** *G. oxydans* 2343 in 1 mL of 100 mM sodium phosphate buffer, pH 7 at 30 °C. The symbols represent conversion (%) of 1,6-HD (filled diamond), and content (%) of 6-HHA (filled square), aldehydes (x) and AA (filled triangle) in the reaction. **D** Conversion (%) of 1,6-HD and content (%) of products formed at 12 h during the oxidation of 10 g/L 1,6-HD by *G. oxydans* strains

Initial tests were performed to compare different *G. oxydans* strains DSM50049, DSM2003, and DSM2343 for their ability to oxidize 1,6-HD (10 g/L) under similar reaction conditions (pH 7, 30 °C) and using same amount of cells based on OD_600_. Figure 1 shows the profiles of 1,6 HD conversion, intermediate (6-HHA) and AA production (Fig. 1, Additional file 1: Scheme S3). Highest conversion rate of 1,6-HD was achieved using *G. oxydans* DSM50049 (Fig. 1A), producing first the intermediate 6-HHA, which was subsequently converted completely to AA within 25 h. Some aldehyde intermediates were also observed during the initial period but did not accumulate (Additional file 1: Figures S1 and S2), indicating that the alcohol oxidizing enzyme(s) activity might be slightly higher than the one for aldehyde oxidation. Additional file 1: Scheme S3 shows the possible pathway for oxidation of 1,6-HD via the different intermediates. In case of *G. oxydans* DSM2003, 6-HHA was obtained as the main product in the initial 12 h, which was subsequently converted rapidly to AA (Fig. 1B). In contrast to *G. oxydans* DSM50049, aldehyde intermediates were not observed, possibly due to the higher expression level or activity of the responsible enzymes. Meanwhile, *G. oxydans* DSM2343 produced more aldehyde intermediates, which were observed during the entire reaction time (Fig. 1C). Accumulation of aldehydes can result in the inhibition of biocatalytic activity and also lead to the loss of cell viability through interaction of the aldehyde carbonyl group with amino- and thiol- groups in proteins and other molecules in the cells [39–41]. These results highlight significant differences in the enzyme makeup of the *G. oxydans* strains, which could be confirmed by analysing their genome sequences. The enzymes involved in the oxidation of 1,6-HD to AA in *G. oxydans* DSM50049 need to be identified.

*Gluconobacter oxydans* DSM50049 was selected for further investigations to improve and optimize the oxidation of 1,6-HD to AA.

### Effect of reaction parameters: cell amount, pH and aeration on oxidation of 1,6-HD to AA by *G. oxydans* cells

**Fig. 2:**
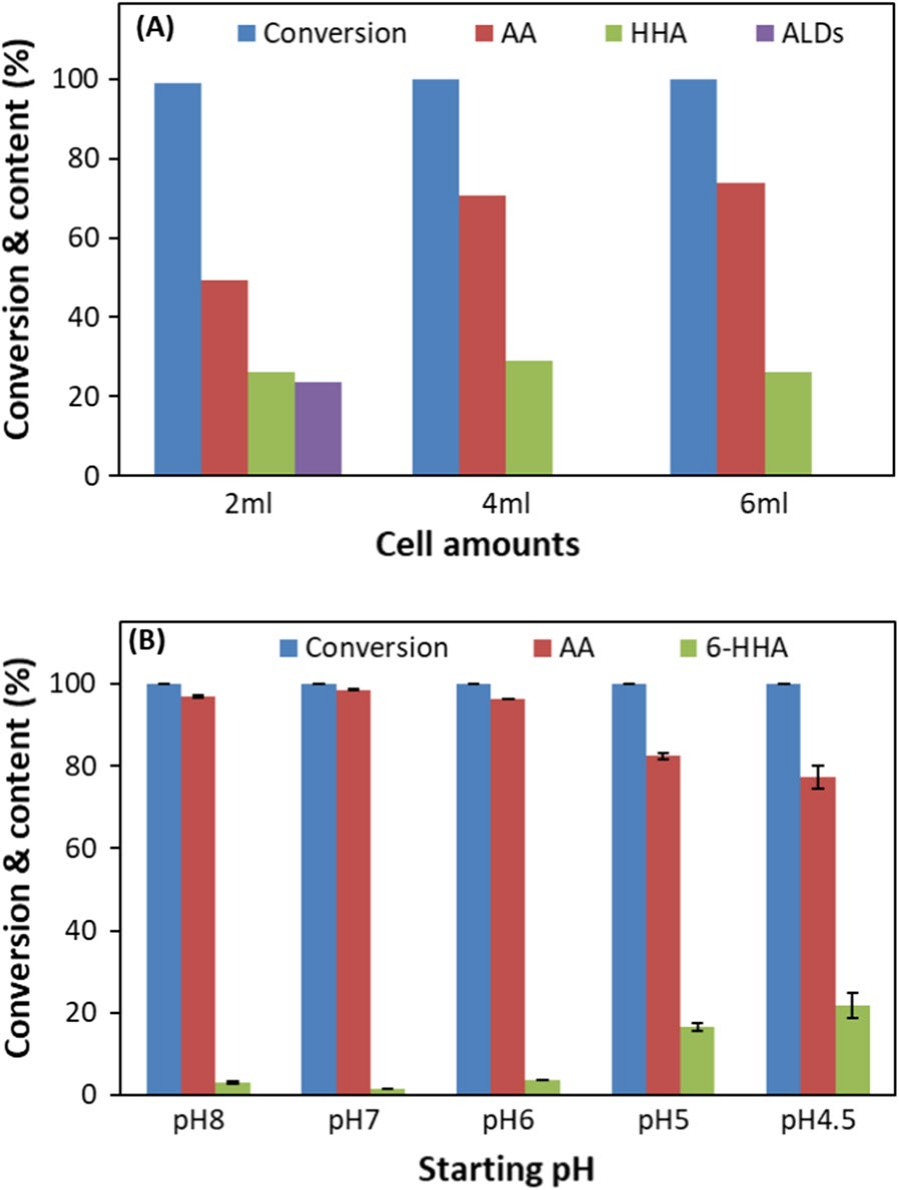
Effect of cell amounts on the oxidation of 10 g/L 1,6-HD to AA via 6-HHA at 30 °C in 1 mL for 12 h by 2 mL (1.6 mg CDW), 4 mL (3.2 mg CDW) and 6 mL (4.8 mg CDW) of *G. oxydans* 50049 in 1 mL of 100 mM sodium phosphate buffer, pH 7 at 30 °C. **A** Effect of cell amount of *G. oxydans* DSM50049, and **B** initial pH on the oxidation of 10 g/L 1,6-HD to AA via 6-HHA in 1 mL at 30 °C. The cell amounts used were 1.6 mg, 3.2 mg and 4.8 mg cell dry weight from 2, 4 and 6 mL culture broth, respectively, in (**A**), and 3.2 mg CDW in (**B**). AA, 6-HHA, and residual 1,6-HD were measured after 12 h in (**A**) and after 24 h in (**B**). pH was not controlled during the reaction

Performing the biotransformation with varying amounts of *G. oxydans* DSM50049 cells revealed complete consumption of 1,6-HD at 12 h in all cases (Fig. 2A). While the product was predominantly a mixture of AA and 6-HHA, the reaction with the lowest cell concentration (1.6 mg CDW per mL) showed significant presence of aldehyde intermediates as the biocatalytic activity for aldehyde oxidation became limiting. Although production and accumulation rate of 6-HHA were higher when using the higher cell amount, the rate and consequent conversion yield to AA were not significantly different. To prevent the accumulation of aldehyde, the cell amount obtained from 4 mL culture equivalent to 3 mg CDW (or more) per milliliter of the reaction would be suitable.

In our previous report, we could control the selective formation of AA and 6-HHA from 1,6-HD by selecting and switching pH value, i.e. 6-HHA was selectively obtained above pH 6, while AA was obtained at pH below 6 as the pH dropped from initial value of pH 7 [20]. The pH was thus considered to be one of the key parameters in biotransformation, hence the reactions with *G. oxydans* DSM50049 were performed at initial pH of 4.5, 5, 6, 7 and 8, respectively, without pH control (Fig. 2B). The conversion of 1,6-HD was rapid and reached over 98% within 18 h in all the cases, but the ratios between 6-HHA and AA were different. At initial pH of 5, the conversion of 6-HHA to AA stopped after 12 h, while 1,6-HD was nearly completely converted to AA at the initial pH of 7 (Fig. 1A). The reaction pH decreased to 4.2 and 4.8 from initial pH of 5 and 7, respectively. With further decrease in initial pH, the amount of AA formed gradually decreased while the 6-HHA amount increased (Fig. 2B), which would be due to a quick drop in the pH. Therefore, the pH control at pH 5–5.5 was necessary to facilitate the conversion of 6-HHA to AA, while 6-HHA was the only product above pH 6 [20].

Investigation of the effect of aeration (0.5–2 L/min, and 20–40% dissolved oxygen) during cultivation of the cells used for 1,6-HD oxidation indicated that higher level of aeration was important for achieving high AA yield (Additional file 1: Figure S3). The DO in the culture grown at fixed air supply dropped to zero before 16 h of cultivation and then went up again to 100% before 24 h as the cells entered stationary phase. Maximum AA concentration of 13.7 g/L was found using cells grown with 20% DO, and further increase in the DO level did not enhance the product yield. Aeration is crucial both for the physiological state of the cells as well as the activity or expression of the responsible enzyme(s) for the oxidation of 1,6-HD to AA. This implies that the drop in oxygen at low aeration rate (0.5–1 L/min) would negatively impact the activity of the cells.

Aeration is a critical factor even for the biotransformation process. The reactions with 20% and 70% DO were compared using cells grown under optimum conditions screened previously. With 20% DO in the reaction, 10 g/L 1,6-HD was totally converted after 19 h, however complete conversion of 6-HHA to AA was not achieved even after 48 h of incubation (Additional file 1: Figure S5). On the other hand, with 70% DO the same amount of 1,6-HD was completely converted within 2 h to 6-HHA, of which > 80% was subsequently converted to AA within 10 h (Fig. 5, first 10 h).

### Cell reusability on the oxidation of 10 g/L 1,6-HD by *G. oxydans* DSM 50049

**Fig. 3:**
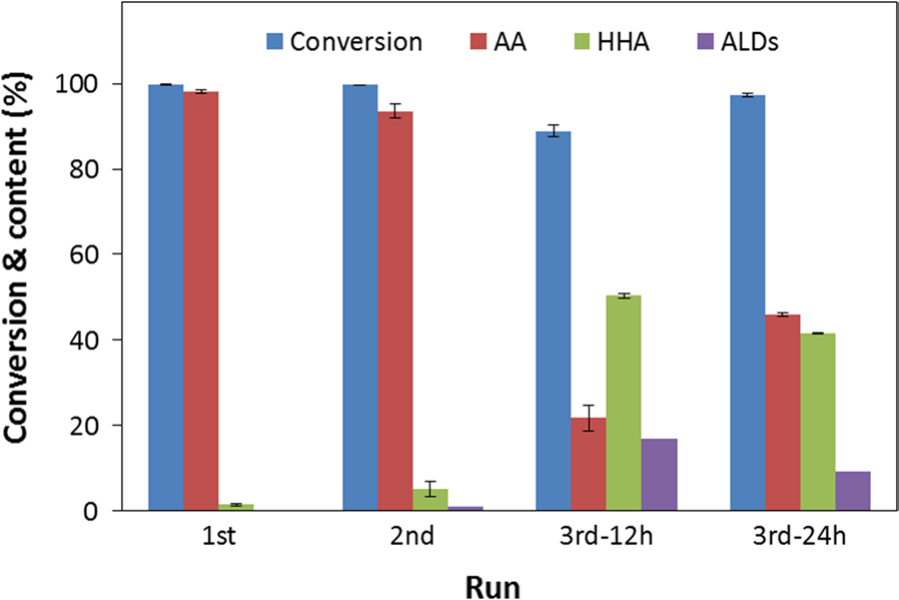
Recycling of *G. oxydans* DSM50049 cells (3.2 mg CDW) for the oxidation of 10 g/L 1,6-HD to AA via 6-HHA in 1 mL of 100 mM sodium phosphate buffer, pH 7 at 30 °C for 24 h (1st and 2nd run)

Recycling of the cells in three consecutive batches for biotransformation of 10 g/L 1,6-HD at starting pH of 7 without pH control for 24 h showed efficient substrate conversion during the first and second runs only, where 100% conversion of 1,6-HD to 6-HHA and over 95% conversion 6-HHA to AA was observed within 24 h (Fig. 3). However, in the third batch the activity of the cells was significantly decreased resulting in a mixture of 3% unconsumed 1,6-HD, 41.7% 6-HHA and 9.4% aldehyde intermediates at 24 h (Fig. 3). The drop in the cell activity could be due to the stress experienced by the cells during recycling, e.g. caused by changes in pH to acidic value during the reaction and then changing to neutral pH to start the next batch. Other factors could be sensitivity to oxygen limitation [38], inhibitory effect of the aldehyde intermediates accumulated in the reaction, and perhaps also to the lack of the cofactor regeneration system. Since the cells are in a resting state, their repair mechanisms are not functioning as it would be for the growing cells.

### Effect of substrate and product concentration on the whole cell oxidation of 1,6-HD and viability of *G. oxydans* cells

**Fig. 4:**
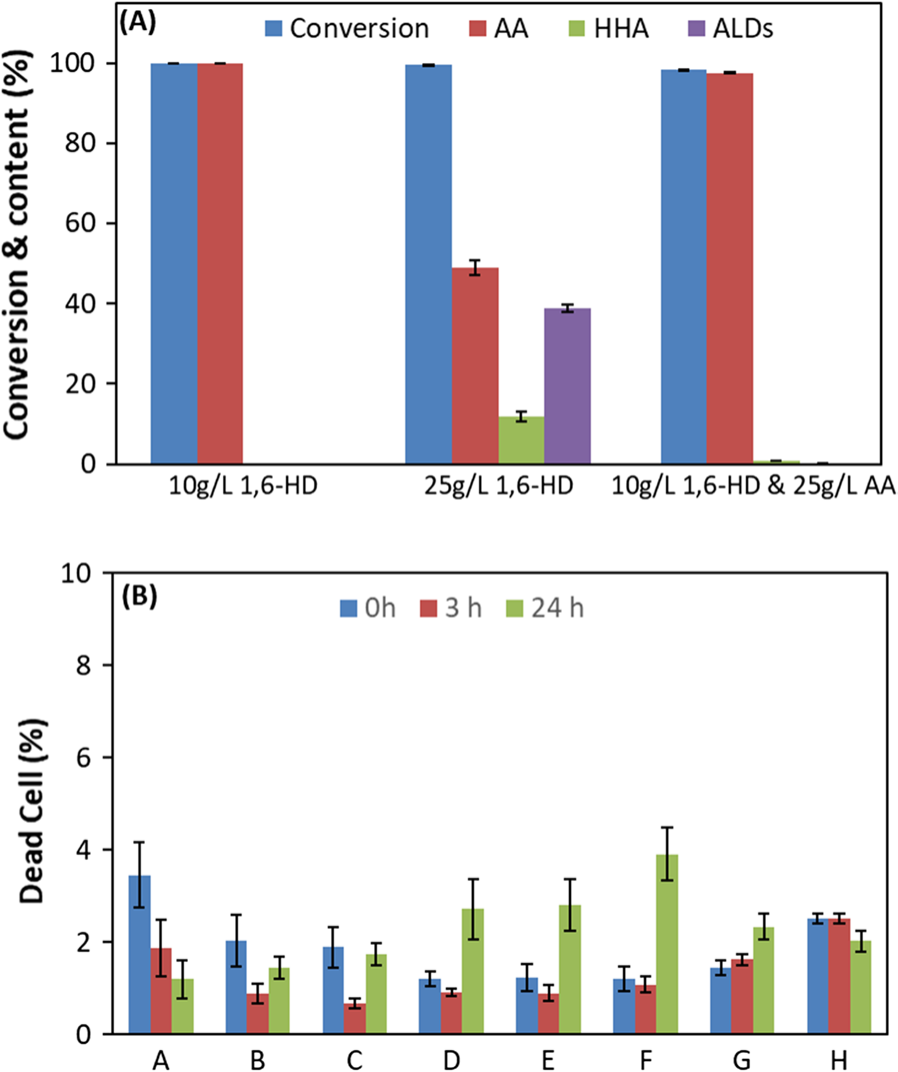
**A** Effect of initial 1,6-hexanediol and adipic acid concentrations on the oxidation of 1,6-HD to AA via 6-HHA in 1 mL at 30 °C for 30 h without pH control by 3.2 mg CDW (obtained from 4 mL cell suspension) of *G. oxydans* 50049. **B** Effect of different starting concentrations of 1,6-HD and AA on the cell viability after 0, 3 and 24 h of oxidation at 30 °C for 30 h without pH control. The initial concentrations of 1,6-HD: AA in g/L were: **A** 0:0, **B** 5:0, **C** 10:0, **D** 15:0, **E** 20:0, **F** 10:10, **G** 10:30, and **H** 10:50

To assess the scalability of the biotransformation, information on the tolerance of *G. oxydans* cells to the substrate and product levels is important. This is also true for the aldehyde intermediates that affect the cell activity and viability negatively [39–41]. Biotransformation of 10 g/L 1,6-HD at an initial pH of 7 and without pH control, resulted in 100% conversion to AA in 30 h at an overall reaction rate of 0.33 g/L h, When 1,6-HD concentration was increased to 25 g/L, over 98% substrate conversion was observed in 30 h, and only 49% AA was formed along with 12% 6-HHA and 39% aldehydes as co-products (Fig. 4A). The low product yield with high amount of intermediates could be due to the pH drop that is consistent with the results in Fig. 2, and/or due to the higher product accumulation and inhibition. To study the effect of the product on the activity of cells, the reaction was supplemented with 25 g/L AA together with the substrate (10 g/L 1.6-HD) at pH 7. Solubility of both substrate and product (tested at 25 g/L each) or (e.g., 110 g AA/L at pH 5.5) was sufficient with pH adjustment under the conditions used (Additional file 1: Figure S4). Complete conversion of 1,6-HD to AA was observed within 30 h. Therefore, the reaction was sensitive to substrate inhibition, while no product inhibition was observed at the tested concentrations, but the pH control was required to complete the reaction.

The effect of product and substrate concentrations was also investigated on cell viability as a function of the cells’ ability to maintain membrane potential needed for functioning of the electron transport chain as well as many transport proteins like ion–ion symports and efflux proteins [42]. A functioning electron transport chain would be essential for the biotransformation as it is needed for the cofactor regeneration [43], and may be affected by high substrate, product or by-product concentration [44]. As shown by flow cytometry measurements, varying the starting concentration of 1,6-HD and AA had little to no effect on the viability of *G. oxydans* cells after 24 h of the reaction (Fig. 4B). The percentage of dead cells was only slightly increased (1–4%) at the end of the reaction in the samples with 15 and 20 g/L 1,6-HD and with 10 g/L 1,6-HD and 10 g/L AA in comparison with the cells in phosphate buffer or saline only (Fig. 4B).

### Fed-batch biotransformation of 1,6-HD to AA by *G. oxydans* DSM 50049

**Fig. 5:**
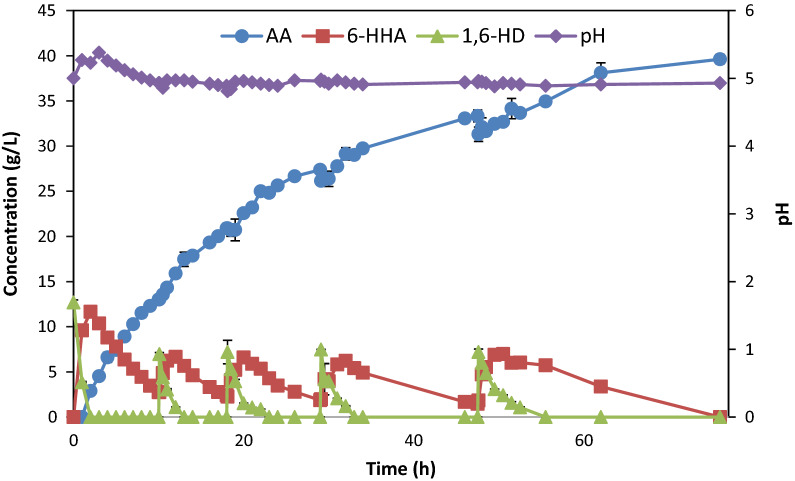
Fed-batch oxidation of 1,6-HD to AA by the resting cells (5.4 g dry weight) of *G. oxydans* DSM 50049 in 1 L scale in 3 L bioreactor at 30 °C, 70% DO (aeration control), and pH controlled at pH 5–5.5. Symbols: accumulated AA concentration (g/L, filled triangle), 6-HHA concentration (g/L, filled square), and 1,6-HD concentration (g/L, filled diamond), and pH (filled circle)

From the overall results above, it was evident that AA production without accumulation of aldehyde intermediates would be facilitated in the pH range of 5–6, high DO level and limiting the 1,6-HD concentration. Hence, the oxidation of 1,6-HD to AA by *G. oxydans* DSM 50049 was performed in a fed-batch mode at 30 °C with pH control at 5–5.5 (Fig. 5, Additional file 1: Figure S5). The reaction was started in a batch mode with 1 L of 10 g/L 1,6-HD with 70% DO, followed by feeding of 1,6-HD into the bioreactor at a concentration of 5 g/L. The transformation of 1,6-HD was maintained at high conversion and yield of AA throughout the entire period of 70 h, with a final titre of approximately 37 g/L. As seen in Fig. 5 and Table 1, the rate of conversion, especially for the second step of the oxidation of 6-HHA to AA, decreased with reaction time.

**Table 1:** Rates of oxidation of 1,6-hexanediol and 6-hydroxyhexanoic acid and production of adipic acid during the fed-batch transformation using *G. oxydans* DSM50049 cells

Kinetic parameters	Batch no and time				
	Batch 1	Batch 2	Batch 3	Batch 4	Batch 5
	0–10 h	10–18 h	18–29 h	29–48 h	48–76 h
^*^q_1,6HDO_ (g/L h)	6.34	3.48	2.49	1.80	1.44
^*^q_6HHA_ (g/L h)	1.11	0.74	0.53	0.27	0.26
^#^q_AA_ (g/L h)	1.31	0.99	0.51	0.42	0.30

^*^Oxidation rate of 1,6HD and 6HHA in g/L h

^#^Production rate of AA in g/L h

The improved performance in the fed-batch transformation compared to the cell reusability experiment may be attributed to maintaining the reaction at pH 5 and minimizing stress to the cells caused by changes in pH during washing and the high-speed centrifugation in between runs. On the other hand, the decrease in the activity rate over time may be due to decrease in enzyme activity and the inability of the cells to maintain the cofactor regeneration over time in the resting cells due to lack of nutrients. Nevertheless, the titre of 37 g/L is among the highest reported for AA from biobased production routes (Table 2). The resulting AA solution obtained from the fed-batch process was subjected to purification. The purified AA was obtained with an overall yield of 74% in a downstream process consisting of cell removal by centrifugation and filtration, concentration, crystallization, washing and drying (Table 3). Significant product losses were observed during washing and vacuum filtration, which can be minimized by better choice of filters and separation procedures.

**Table 2:** Comparison of titers of adipic acid or its intermediate muconic acid obtained by different microbial production routes

Substrate	Organism	Intermediate	Titer (g/L)	References
*p-*Coumarate	*Pseudomonas putida*	Muconic acid (can be converted to AA with 100% yield)	13.5	[12]
Glucose	*Escherichia coli*	Muconic acid	36.8	[13]
Glucose	*Escherichia coli*	Direct from glucose	36·10^−3^	[14]
Glycerol	*Escherichia coli*	Direct from glucose	2.5	[20]
Glucose	*Thermobifida fusca*	Direct from glucose	2.23	[16]
Coconut oil	*Yeast*		52	[17]
1,6-Hexandiol	*Gluconobacter oxydans*		40	This work

**Table 3:** Purification of adipic acid from the end product of the fed-batch process of 1,6-hexanediol oxidation in 1 L reaction volume using the resting cells of *G. oxydans* DSM 50049 at pH 5.2

Purification step	AA step yield (%)	AA overall yield (%)
Cell removal	94	94
Concentration and crystallisation	94.7	89
Washing and drying	83.1	74

Initial adipic acid concentration: 40 g/L

## Conclusion

A facile and efficient process was demonstrated for the production of adipic acid from 1,6-hexanediol, a potential biobased diol obtained from 5-HMF. The selective microbial oxidation of diol to dicarboxylic acid can be scalable without substrate and product inhibition by fed-batch process with pH-control. The results presented here provide a basis for an alternative facile process to produce AA from renewable resources without notable greenhouse gas emissions. Also, identification of the responsible enzymes, presently under investigation, will be a valuable step for further improvement of the microbial biocatalyst and the process.

## Materials

1,6-Hexanediol (1,6-HD), 6-hydroxyhexanoic acid (6-HHA) and adipic acid (AA), were procured from Sigma-Aldrich. The bacterial strains, *Gluconobacter oxydans* DSM 50049, *G. oxydans* DSM 2343 and *G. oxydans* DSM 2003 were obtained from Deutsche Sammlung von Mikroorganismen und Zellkulturen GmbH (Braunschweig, Germany). Yeast extract, glycerol, sodium dihydrogen phosphate and disodium hydrogen phosphate were purchased from Merck. All chemicals were used without further treatment.

### Cultivation of *Gluconobacter oxydans*

Lyophilized cells of *G. oxydans* DSM 50049, 2343 and 2003 were individually inoculated into 50 mL *Gluconobacter* broth medium in 250 mL flasks, containing (per liter) 100 g glucose and 10 g yeast extract at pH 6.8. The flasks were incubated in a shaker incubator (Ecotron, Infors HT, UK) at 30 °C and 200 rpm for 24 h. Glycerol stocks (20%) of the bacterial cultures were prepared and stored at − 20 °C for further use [20].

One hundred microliter glycerol stock of *G. oxydans* was inoculated into 50 mL medium in a 250 mL flask, containing per liter: 25 g glycerol and 10 g yeast extract with pH adjusted to 5, and incubated as described above. Thereafter, the culture broth was centrifuged at 4700×*g* for 15 min (Sorvall LYNX 4000, Thermo Scientific, Germany), the cell pellet was separated and washed twice using 100 mM sodium phosphate buffer pH 7 prior to use in the oxidation reactions.

Cultivation of *G. oxydans* 50049 for studying the effect of aeration was performed in 1 L glycerol containing culture medium in a 3 L bioreactor (Applikon, Microbial Biobundle, The Netherlands). The oxygen level was controlled at fixed aeration rates of 0.5, 1, 1.5 and 2 vvm with a stirring rate of 500 rpm, and alternatively with a fixed aeration rate of 1 vvm and adjustable stirring rate controlled by the DO level of 20% or 40%. Each bioreactor was inoculated with 25 mL of overnight culture of *G. oxydans* 50049, and cultivation was performed for 24 h at pH 5 and 30 °C. The collected cell culture was centrifuged at 4700×*g* for 15 min (Sorvall LYNX 4000, Thermo Scientific, Germany), then washed with 100 mM sodium phosphate buffer pH 7, and re-centrifuged before being used to oxidize 1,6-HD.

For use in the fed-batch process of 1,6-HD oxidation, *G. oxydans* cells were produced in 22 L glycerol medium in a 30 L bioreactor (Bioengineering AG, Switzerland), inoculated with 550 mL culture prepared in the same way as described above, and performing the cultivation for 18 h at 30 °C, 500 rpm, and pH controlled at 5 by addition of 5 M NaOH solution. DO concentration was maintained at 40% by sparging 22 L/min air at the start of the cultivation and later by a manual adjustment of stirrer speed and airflow. The cells were collected by centrifugation at 8500×*g* and 4 °C for 20 min (Sorvall, Thermo Scientific, Germany), the cell pellet washed once with 500 mL of 100 mM sodium phosphate buffer pH 7, and re-centrifuged.

### Batch biotransformation of 1,6-HD using* Gluconobacter oxydans* cells

Oxidation of 1,6-HD by *G. oxydans* in batch mode was evaluated at two different volumes. The cell pellet (3 mg dry weight) of *G. oxydans* collected from 4 mL cultivation medium (OD 2.5), was re-suspended in 1 mL of 100 mM sodium phosphate buffer at a predefined pH, supplemented with a given concentration of 1,6-HD in 4 mL vials that were incubated in a thermomixer (MKR 13, HLC Biotech, Germany) at 30 °C and 500 rpm without pH control. Twenty microliter samples were collected during the reaction for analyzing substrate and product concentrations.

The cell pellet from cultures with varied aeration was resuspended in 10 mL of 100 mM sodium phosphate buffer at pH 7 supplemented with 10 mg/mL 1,6-HD, in a 50 mL bottle covered with a cotton plug stopper, and placed in a shaker incubator (Ecotron, Infors HT, UK) at 200 rpm and 30 °C without pH control. One millilitre samples were collected from the reaction for HPLC analysis.

### Fed-batch biotransformation of 1,6-HD by *G. oxydans* DSM 50049

The cell pellet (around 5.4 g dry weight) obtained from 4  L culture broth was re-suspended in 1 L of 100 mM sodium phosphate buffer, pH 7, supplemented with 10 g/L 1,6-HD in a 3 L bioreactor (Applikon), and the biotransformation was performed at 30 °C, 500 rpm, with pH being continuously maintained at 5 using 5 M NaOH solution. Dissolved oxygen was maintained at 70% during the entire experiment by regulating the stirrer speed. Fifty microliters of approximate 100 g/L 1,6-HD solution (giving an approximate concentration of 5 g/L in the reaction volume) were fed at 10, 18, 29, and 47.5 h of the reaction. Seven millilitre samples were collected during the reaction for analyzing substrate and product concentrations.

### Recovery and purification of adipic acid

The purification of AA was performed using the solution (approximately 1 L volume) obtained from fed-batch biotransformation of 1,6-HD. The pH of solution was adjusted to 9 with 5 M NaOH followed by centrifugation at 4000×*g* and 4 °C for 20 min, and vacuum filtration using a filter with 0.4 µm pore size for removing any cell debris left after centrifugation. AA was precipitated and recovered from the filtrate by adjusting the pH to 1 using the concentrated 36% HCl solution, (Alfa Aesar, Haverhill, MA, USA). The filtrate was further concentrated to 100 mL to recover the remaining soluble AA through water removal using a rotary evaporator (Rotavapor R-300, Büchi, Germany). The resulting 100 mL AA solution was kept overnight at 4 °C for AA precipitation. Thereafter, another centrifugation was done at 4000×*g* and 4 °C for 20 min to separate the AA crystals from the solution. For further purification, the recovered AA crystals were dissolved at 95 °C in 100 mL MilliQ water and kept at 4 °C overnight for AA precipitation. The precipitated AA was recovered and washed with 200 mL cold MilliQ water through filtration under reduced pressure. The purified AA powder was obtained after drying in oven at 50 °C for 24 h.

### Analytical procedures

Cell density was determined by measuring the optical density of the cell broth at 620 nm using UV/Vis spectrophotometer (Ultrospec 1000, Pharmacia Biotech, Sweden). The cell dry weight (CDW) was determined by collecting cells from 1 mL fermentation broth at 4700×*g* for 10 min in a dried pre-weighed 1.5 mL Eppendorf tube. The collected cell pellet was dried overnight at 105 °C. The increase in weight of the tube equals CDW per millilitre. The OD_620_ was correlated to CDW by the following equation:

1 $${\text{CDW }}\left( {{\text{g L}}^{{ - {1}}} } \right) = {\text{OD}}_{{{62}0}} \times 0.{3}$$

Quantitative analyses of reaction components were performed using gas chromatography (GC, Varian 430-GC, Varian, USA) equipped with FactorFour Capillary column, VF-1 ms (Varian, 15 M × 0.25 mm) and a flame ionization detector. The initial column oven temperature was increased from 50 to 250 °C at a rate of 20 °C/min. The samples, diluted with acetonitrile (0.1% DMSO as external standard), to a sample concentration of 0.1–0.5 mg/mL, were injected in split injection mode of 10% at 275 °C. Conversion of the substrates and concentration of products formed were calculated from the standard curves on the gas chromatograms.

The concentrations of 1,6-HD, 6-HHA and AA from the fed-batch experiment were determined using HPLC with a BioRad Aminex HPX87H (Fast Acid) column (BioRad, Hercules, CA, USA) maintained at 65 °C and refractive index detector. Sulfuric acid (0.5 mM) was used as the mobile phase at a flow rate of 0.6 mL/min. The different compounds were identified and quantified by using an external standard.

Structures of the products were elucidated by gas chromatography–mass spectrometry (GC–MS, 431-GC and 210-MS, Varian, USA) run under the same conditions as the GC analysis described above.

The selectivity of the process was calculated from amounts (mmol) of product formed and consumed substrate (Selectivity (%) = (product (mmol)/consumed substrate (mmol)) × 100). Isolated yields of the products were calculated according to the molar ratio of isolated products to used substrates for representative reactions.

Flow cytometry was used to check the extent of live, dead and damaged cells of *G. oxydans* during oxidation of varying initial concentrations of 1,6-HD to AA. Cells were taken at 0, 3 and 24 h from the reactions by centrifugation and resuspended in 100 mM PBS buffer to an OD_600_ of 0.04 before staining with the dyes SYBR™ Green (Bio-rad, Hercules, California, USA), (diluted 10,000 times according to the supplier’s specifications) and propidium iodide (1 µg/mL) to estimate the live and dead cells, respectively. The cell suspension was stained for 10 min on ice and the measurement was done in a BD Accuri C6 flow cytometer Plus (San Jose, CA, USA). Cells killed by heat treatment at 100 °C for 30 min were used to create the grouping of dead cells, while freshly grown cells were used to group the live cells.

## Supplementary Information

**Additional file 1: Scheme S1. **Commercial adipic acid production from petroleum-derived benzene through cyclohexane by chemical catalysis, and its use in the production of Nylon 6,6. Oxidation of cyclohexane using Co catalyst (cobalt- (II) naphthenate) and air as an oxidant to KA oil (ketone-alcohol oil), a mixture of cyclohexanone and cyclohexanol, is typically conducted at low conversions (3 to 8%) to maintain high selectivity (70–90%), necessitating extensive feed recycling and huge capital costs [19]. Further oxidation of KA oil to adipic acid occurs under harsh conditions using nitric acid, with coproduction of undesired N_2_O. **Scheme S2.** An integrated microbial-chemical route for the production of biobased 6hydroxyhexanoic acid (6-HHA), adipic acid (AA), and ε-caprolactone (ε-CL) via 5hydroxymethylfurfural (5-HMF) and 1,6-hexanediol (1,6-HD) [20]. **Scheme S3. **Possible oxidation pathway of 1,6-hexanediol to adipic acid via different oxidative intermediates. **Figure S1. **GC chromatograms on the 1,6-HD oxidation to AA at time 6, 12 h and 30 h by *G. oxydans* 50049. 1. hexan-dial, 2. 6-hydroxyhexanal, 3. 1,6-hexanediol, 4. 6oxohexanoic acid, 5. 6-hydroxyhexanoic acid, 6. Adipic acid. **Figure S2.** Mass data determined by GC–MS on the microbial oxidation of 10 g/L 1,6hexanediol (1,6-HD) to adipic acid (AA). The numbered compounds in Figure S1 were confirmed by Mass; 1. hexan-dial, 2. 6-hydroxyhexanal, 3. 1,6-hexanediol, 4. 6oxohexanoic acid, 5. 6-hydroxyhexanoic acid, 6. Adipic acid. **Figure S3. **Effect of aeration on the oxidation of 10 g/L 1,6-hexanediol to adipic acid at 30 °C for 24 h without pH control by *G. oxydans* 50049. (A) With the cells grown under different aeration conditions either through sparging of different air volumes (0.5–2 L/min) or with fixed DO (20–40%) by controlling the stir rate. **Figure S4.** Solubility of adipic acid in 0.1 M PBS at pH 5.5, 5 and 4.5; In 5 mL 0,1 M PBS buffer (pH 8,3) 1500 or 2000 mg of adipic acid was resuspended and the pH was adjusted with 5 M NaOH to pH 4.5 and 5 for the 150 mg adipic acid and to pH 5 for the 2000 mg. The volume was adjusted to 10 mL with PBS buffer of respective pH and the solution incubated at 30 °C for 2 h to allow the adipic acid saturate the buffer. **Figure S5. **Parameters for fed-batch biotransformation of 1,6-HD to AA in 1 L working volume in 3 L bioreactor.
