# Supplementary material for: A facile process for adipic acid production in high yield by oxidation of 1,6-hexanediol using the resting cells of Gluconobacter oxydans
Source: Microb Cell Fact. 2022 Oct 28;21:223. doi: 10.1186/s12934-022-01947-6 (PMC9617331; doi:10.1186/s12934-022-01947-6)

Supplementary material

A facile process for adipic acid production in high yield by oxidation of 1,6-hexanediol using the resting cells of *Gluconobacter oxydans*

Sang-Hyun Pyo[1,*], Mahmoud Sayed[1,2], Oliver Englund Örn[1], Jorge Amorrortu Gallo[1,†], Nídia Fernandez Ros[1,†], and Rajni Hatti-Kaul[1]

[1]Division of Biotechnology, Department of Chemistry, Center for Chemistry and Chemical Engineering, Lund University, SE-22100 Lund, Sweden

[2] Department of Botany and Microbiology, Faculty of Science, South Valley University, 83523 Qena, Egypt

[†]Equal contribution by authors

* *Corresponding author*

Tel: +46-46-222-4838; Fax: +46-46-222-4713

E-mail: Sang-Hyun.Pyo@biotek.lu.se (S.-H. Pyo)

Scheme S1. Commercial adipic acid production from petroleum-derived benzene through cyclohexane by chemical catalysis, and its use in the production of Nylon 6,6. Oxidation of cyclohexane using Co catalyst (cobalt- (II) naphthenate) and air as an oxidant to KA oil (ketone-alcohol oil) , a mixture of cyclohexanone and cyclohexanol, is typically conducted at low conversions (3 to 8 %) to maintain high selectivity (70−90 %), necessitating extensive feed recycling and huge capital costs [19]. Further oxidation of KA oil to adipic acid occurs under harsh conditions using nitric acid, with co-production of undesired N_2O.

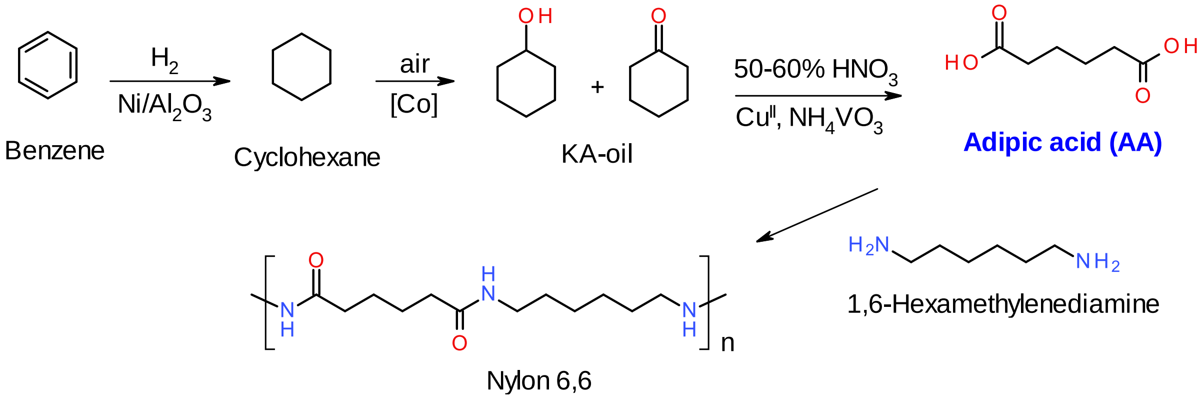

Scheme S2. An integrated microbial-chemical route for the production of biobased 6-hydroxyhexanoic acid (6-HHA), adipic acid (AA), and ε-caprolactone (ε-CL) via 5-hydroxymethylfurfural (5-HMF) and 1,6-hexanediol (1,6-HD) (21).

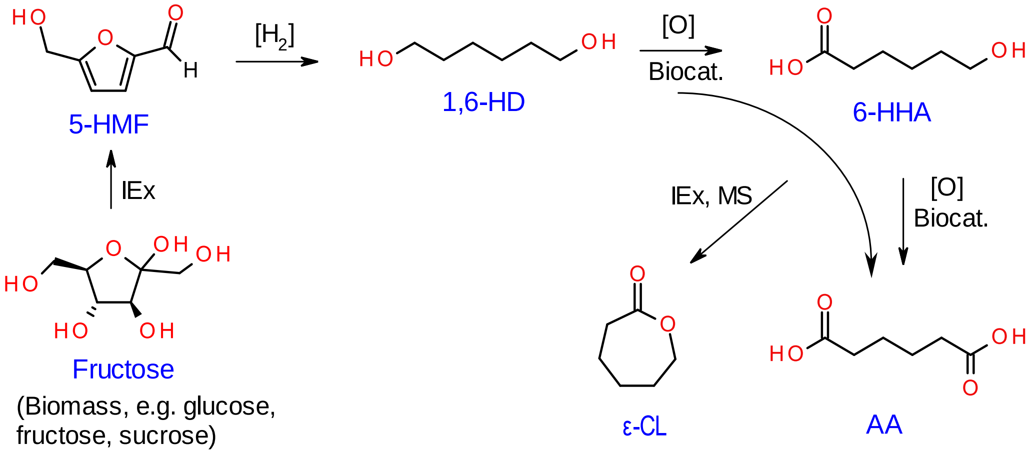

Scheme S3. Possible oxidation pathway of 1,6-hexanediol to adipic acid via different oxidative intermediates.

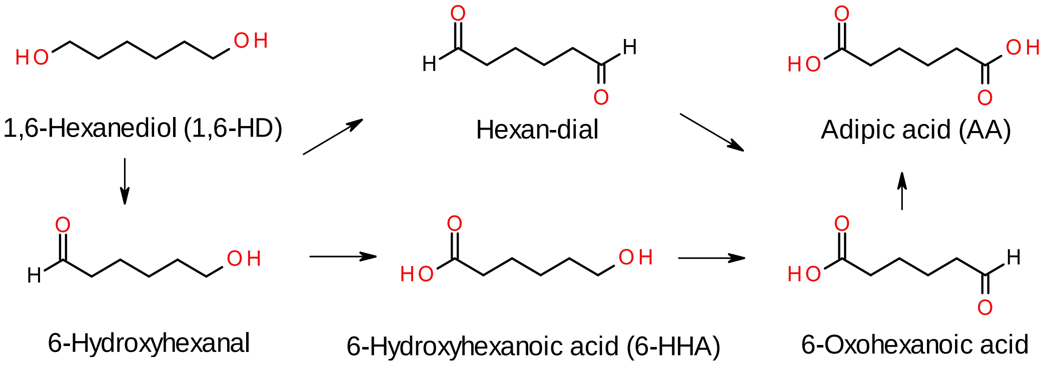

Figure S1. GC chromatograms on the 1,6-HD oxidation to AA at time 6, 12 h and 30 h by *G. oxydans* 50049. 1. hexan-dial, 2. 6-hydroxyhexanal, 3. 1,6-hexanediol, 4. 6-oxohexanoic acid, 5. 6-hydroxyhexanoic acid, 6. Adipic acid.

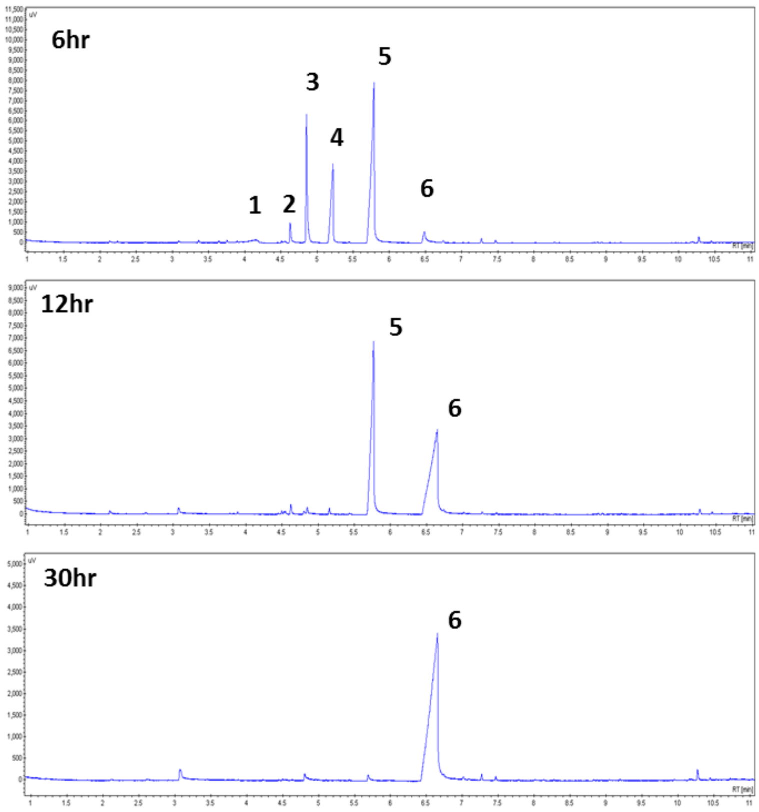

Figure S2. Mass data determined by GC-MS on the microbial oxidation of 10 g/L 1,6-hexanediol (1,6-HD) to adipic acid (AA). The numbered compounds in Figure S1 were confirmed by Mass; 1. hexan-dial, 2. 6-hydroxyhexanal, 3. 1,6-hexanediol, 4. 6-oxohexanoic acid, 5. 6-hydroxyhexanoic acid, 6. Adipic acid.

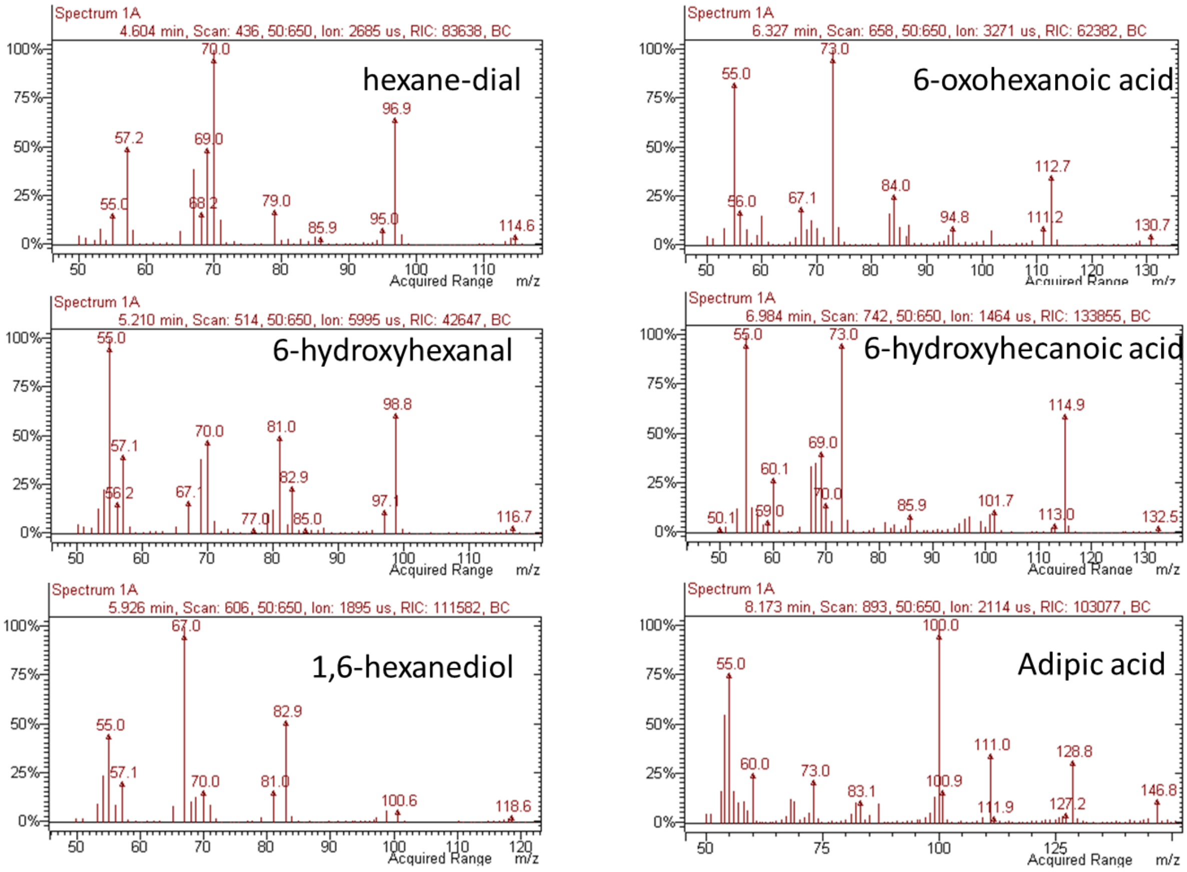

Figure S3. Effect of aeration on the oxidation of 10 g/L 1,6-hexanediol to adipic acid at 30 °C for 24 h without pH control by *G. oxydans* 50049. (A) With the cells grown under different aeration conditions eihter through sparging of different air volumes (0.5-2 L/min) or with fixed DO (20-40 %) by controling the stir rate.

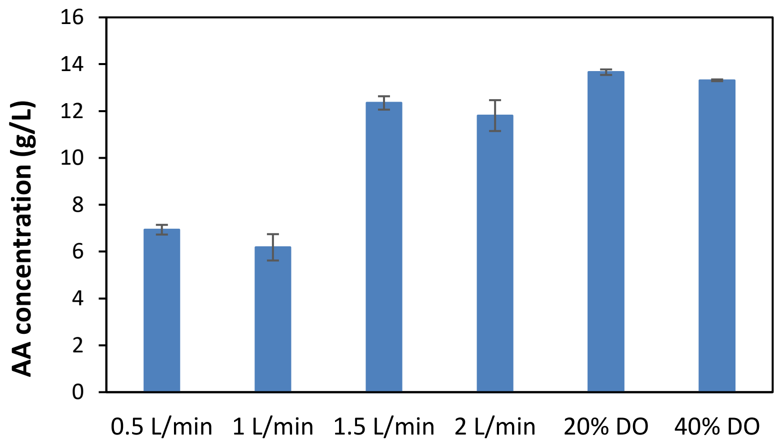

Figure S4. Solubility of adipic acid in 0.1 M PBS at pH 5.5, 5 and 4.5; In 5 ml 0,1 M PBS buffer (pH 8,3) 1500 or 2000 mg of adipic acid was resuspended and the pH was adjusted with 5 M NaOH to pH 4.5 and 5 for the 150 mg adipic acid and to pH 5 for the 2000 mg. The volume was adjusted to 10 ml with PBS buffer of respective pH and the solution incubated at 30°C for 2 hr to allow the adipic acid saturate the buffer.

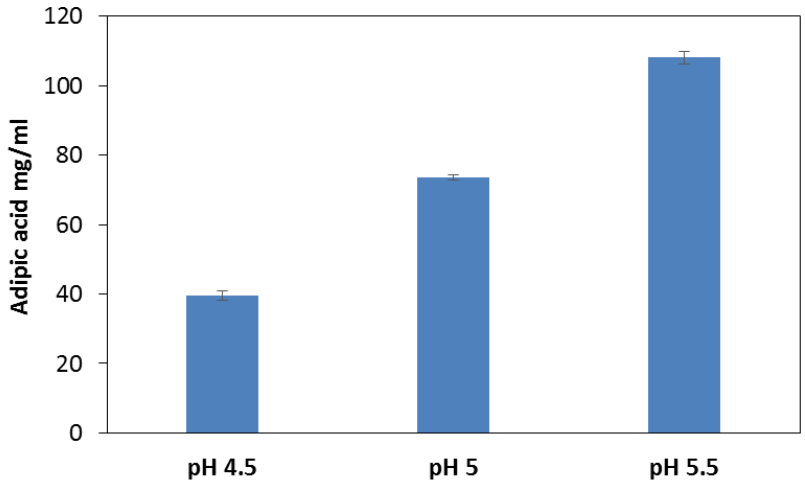

Figure S5. Parameters for fed-batch biotransformation of 1,6-HD to AA in 1L working volume in 3L bioreactor

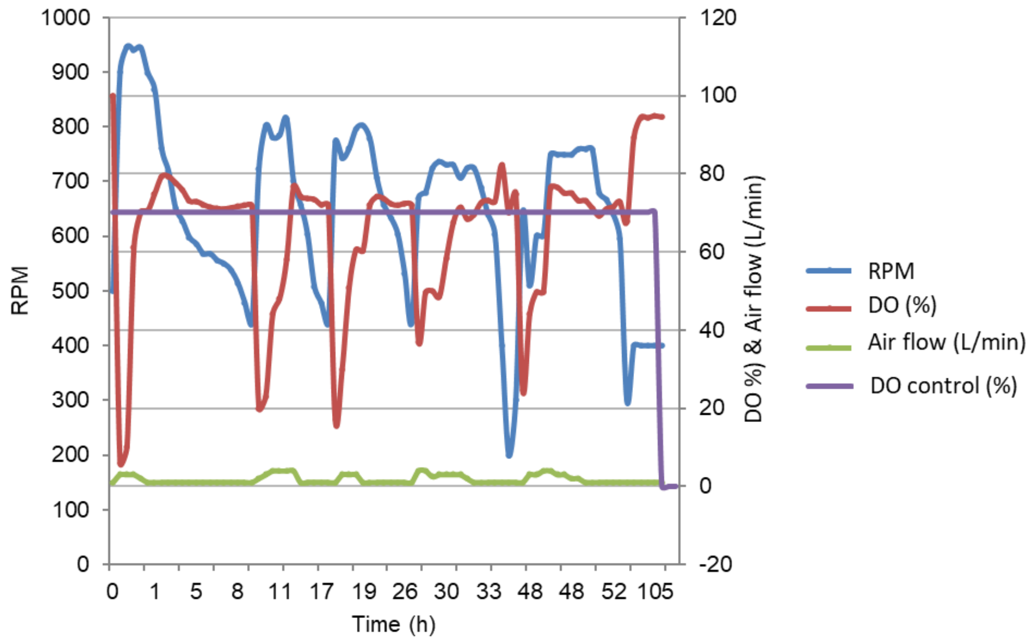

Supplement: Supplementary file 1 — Additional file 1: Scheme S1. Commercial adipic acid production from petroleum-derived benzene through cyclohexane by chemical catalysis, and its use in the production of Nylon 6,6. Oxidation of cyclohexane using Co catalyst (cobalt- (II) naphthenate) and air as an oxidant to KA oil (ketone-alcohol oil), a mixture of cyclohexanone and cyclohexanol, is typically conducted at low conversions (3 to 8%) to maintain high selectivity (70–90%), necessitating extensive feed recycling and huge capital costs [19]. Further oxidation of KA oil to adipic acid occurs under harsh conditions using nitric acid, with coproduction of undesired N2O. Scheme S2. An integrated microbial-chemical route for the production of biobased 6hydroxyhexanoic acid (6-HHA), adipic acid (AA), and ε-caprolactone (ε-CL) via 5hydroxymethylfurfural (5-HMF) and 1,6-hexanediol (1,6-HD) [20]. Scheme S3. Possible oxidation pathway of 1,6-hexanediol to adipic acid via different oxidative intermediates. Figure S1. GC chromatograms on the 1,6-HD oxidation to AA at time 6, 12 h and 30 h by G. oxydans 50049. 1. hexan-dial, 2. 6-hydroxyhexanal, 3. 1,6-hexanediol, 4. 6oxohexanoic acid, 5. 6-hydroxyhexanoic acid, 6. Adipic acid. Figure S2. Mass data determined by GC–MS on the microbial oxidation of 10 g/L 1,6hexanediol (1,6-HD) to adipic acid (AA). The numbered compounds in Figure S1 were confirmed by Mass; 1. hexan-dial, 2. 6-hydroxyhexanal, 3. 1,6-hexanediol, 4. 6oxohexanoic acid, 5. 6-hydroxyhexanoic acid, 6. Adipic acid. Figure S3. Effect of aeration on the oxidation of 10 g/L 1,6-hexanediol to adipic acid at 30 °C for 24 h without pH control by G. oxydans 50049. (A) With the cells grown under different aeration conditions either through sparging of different air volumes (0.5–2 L/min) or with fixed DO (20–40%) by controlling the stir rate. Figure S4. Solubility of adipic acid in 0.1 M PBS at pH 5.5, 5 and 4.5; In 5 mL 0,1 M PBS buffer (pH 8,3) 1500 or 2000 mg of adipic acid was resuspended and the pH was adjusted [file 12934_2022_1947_MOESM1_ESM.pdf]
